# Serum bile acids in liver cirrhosis promote neutrophil dysfunction

**DOI:** 10.1002/ctm2.735

**Published:** 2022-02-27

**Authors:** Irina Balazs, Angela Horvath, Bettina Leber, Nicole Feldbacher, Wolfgang Sattler, Florian Rainer, Günter Fauler, Sonja Vermeren, Vanessa Stadlbauer

**Affiliations:** ^1^ Department of Internal Medicine Division of Gastroenterology and Hepatology Medical University of Graz Graz Austria; ^2^ Center for Biomarker Research in Medicine (CBmed) Graz Austria; ^3^ Department of Surgery Division of Transplantation Surgery Medical University of Graz Graz Austria; ^4^ Gottfried Schatz Research Center (for Cell Signaling, Metabolism and Aging) Division of Molecular Biology and Biochemistry Medical University of Graz Graz Austria; ^5^ Center for Explorative Lipidomics BioTechMed Graz Graz Austria; ^6^ Clinical Institute of Medical and Chemical Laboratory Diagnostics Medical University of Graz Graz Austria; ^7^ Centre for Inflammation Research, Institute for Regeneration and Repair University of Edinburgh Edinburgh UK

1

Dear Editor,

Neutrophil dysfunction is common in cirrhotic patients^1^ and is associated with mortality.^2^ Given the highly increased bile acid (BA) levels in liver cirrhosis^3^ and their previously shown anti‐inflammatory effects,^4^, ^5^ we studied if BAs contribute to the development of cirrhosis‐associated neutrophil dysfunction.

We analysed phagocytosis and reactive oxygen species (ROS) production of neutrophils and serum BAs in cirrhotic patients (*n* = 109) and healthy controls (*n* = 21) (Table 1, Tables S3–S4, Supplementary Material and Methods). The cirrhotic cohort had a larger proportion of non‐phagocytosing neutrophils in whole blood (Figure S1A,B) and dysregulated ROS production with more neutrophils from cirrhotics producing ROS without a stimulus or in response to N‐formyl‐met‐leu‐phe (fMLF), and less neutrophils from cirrhotics producing ROS in response to *E. coli* compared to healthy controls (Figure S1C–E). The level of intracellular ROS produced by these neutrophils was not significantly different between the groups (Figure S2A–C).

**TABLE 1:** Demographic characteristics and liver function parameters of cirrhotic patients depending on liver cirrhosis aetiology (median ± interquartile range)

Characteristic	Alcoholic (*n* = 54)	HCV (*n* = 32)	Other (*n* = 23)	*p*‐value
Age (years)	56 ± 12	60 ± 9	55 ± 14	n.s.
Sex (Male/Female, n)	42/12	22/10	13/10	n.s.
Child‐Pugh group (A/B+C, n)	37/17	25/7	17/6	n.s.
Child‐Pugh score	6 ± 2	5 ± 1	5 ± 2	n.s.
MELD score	12 ± 6	8 ± 4	10 ± 7	*p *= .014^a^
AST (U/l)	44 ± 26	91 ± 72	40 ± 38	*p *< .001^a^, ^b^
ALT (U/l)	32.5 ± 19	70 ± 80	34 ± 38	*p *< .001^a^, *p *= .001^b^
GGT (U/l)	105.5 ± 176	114.5 ± 135	120.5 ± 285	n.s.
AP (U/l)	109 ± 74	94 ± 45	105 ± 100	n.s.
Bilirubin (mg/dl)	1.5 ± 1.6	.9 ± .9	1.1 ± 3.5	*p *= .030^a^
INR (ratio)	1.3 ± .3	1.1 ± .3	1.2 ± .3	*p *= .030^c^
Creatinine (mg/dl)	.8 ± .3	.8 ± .2	.8 ± .3	n.s.
Albumin (g/dl)	4.0 ± 1.1	4.0 ± .8	4.5 ± 1.0	n.s.
Neutrophils (x10^9^/l)	2.9 ± 1.4	2.6 ± 1.0	3.1 ± 2.8	n.s.

*Note*: Median ± interquartile range. Group ‘Other’ included patients with alpha‐1 antitrypsin deficiency (*n* = 1), secondary sclerosing cholangitis in critically ill patients (*n* = 2), hepatitis B (*n* = 4), haemochromatosis (*n* = 2), non‐alcoholic fatty liver disease (*n* = 5), medication associated (*n* = 2), Wilson disease (*n* = 3) and cryptogenic cirrhosis (*n* = 4). ALT, alanine transaminase; AP, alkaline phosphatase; AST, aspartate transaminase; GGT, gamma‐glutamyl transferase; HCV, hepatitis C virus; INR, international normalized ratio; MELD, model for end‐stage liver disease; n.s., not significant.

^a^ Between HCV and alcoholic;.

^b^ Between HCV and other;.

^c^ Between other and alcoholic groups.

Serum BA composition was also different in cirrhotic patients compared to healthy controls (Figures S3A,B and S4A,B). The relative abundance of total deoxycholic acid (DCA) in cirrhotic patients was lower than in healthy controls (Figure S3C, Table S5). Secondary, unconjugated and glycine‐conjugated BAs were significantly less abundant in cirrhotic patients’ sera compared to healthy sera. Taurine‐conjugated BAs were significantly more abundant in cirrhotic sera compared to healthy sera (Figure S3D–G, Table S5).

Neutrophil function and BA composition were significantly different between the groups according to liver cirrhosis severity and aetiology as shown by univariate (Figures S5A–E and S6A–E, Tables S6 and S7) and multivariate analyses (Figures S7A–D, S8A–F and S9A–F). The phagocytic capacity of neutrophils was lowest in hepatitis C patients (Figure S6B), who had the highest relative abundance of total chenodeoxycholic acid (CDCA) in serum (Table S7).

Further analysis showed that higher total CDCA relative abundance and lower ursodeoxycholic acid (UDCA) relative abundance could significantly predict the decreased neutrophil phagocytosis in cirrhotic patients. Lower UDCA relative abundance could also predict the dysfunctional ROS production in response to *E. coli* (Figure S10A–C, Table S8).

In order to validate the findings observed with clinical samples in vitro, we treated neutrophils from healthy donors with an artificial ‘BA mix’ that mimicked the BA composition in cirrhotic cohort, as well as with individual or total (sum of unconjugated, taurine and glycine conjugated forms) BAs in pathophysiologically relevant concentrations (Figures 1A, 2A and 3A, Tables S1 and S2, Supplementary Material and Methods). Cytotoxicity of BAs was ruled out (Figure S11).

**FIGURE 1:**
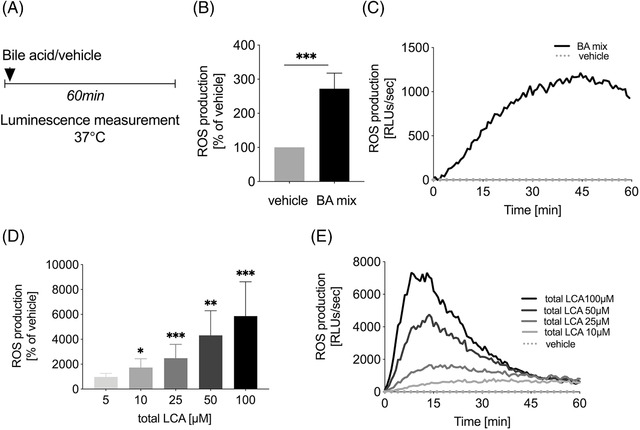
Total LCA induce ROS production by unstimulated neutrophils. (A) Experimental setup. (B–E) ROS production of isolated healthy donor neutrophils after treatment with bile acid (BA)/vehicle. (C and E) Representative examples and (B and D) total ROS production over time. ROS data were normalised and compared to vehicle‐treated control (PBS) and represent a minimum of four separate experiments. Data are presented for bile acids, which significantly influenced ROS production; error bars, SEM; *p < .05, **p < .01, ***p < .001 (unpaired *t*‐test). BAs, bile acids; LCA, lithocholic acid; PBS, phosphate‐buffered saline; RLUs, relative light units; ROS, reactive oxygen species; SEM, standard error of the mean

In our experiments ‘BA mix’ significantly induced ROS production by unstimulated neutrophils (Figure 1B,C). ROS production in response to fMLF or *E. coli*, however, was not changed after treatment with ‘BA mix’ (Figure S14A,B). Further experiments with individual BAs revealed that total LCA, but not other BAs, caused increased ROS production (Figure 1D,E, Figure S12A), with glycolithocholic acid having the most pronounced effect (Figure S13A). LCA has been shown to bind Takeda G protein‐coupled Receptor 5, whose activation can trigger ROS production,^6^, ^7^ which may explain the effects of LCA and its conjugates.

Total CDCA and total LCA reduced ROS production in response to fMLF (Figure 2B,D,F), but not their unconjugated and conjugated forms individually (Figure S13B,C). CDCA has been previously described to inhibit neutrophil chemotaxis and calcium flux, via competing with fMLF for binding to the Formyl Peptide Receptor 1 (FPR1).^8^ The decreased ROS production caused by pre‐incubation with total LCA might be a consequence of cell exhaustion following the LCA‐mediated ROS release.

**FIGURE 2:**
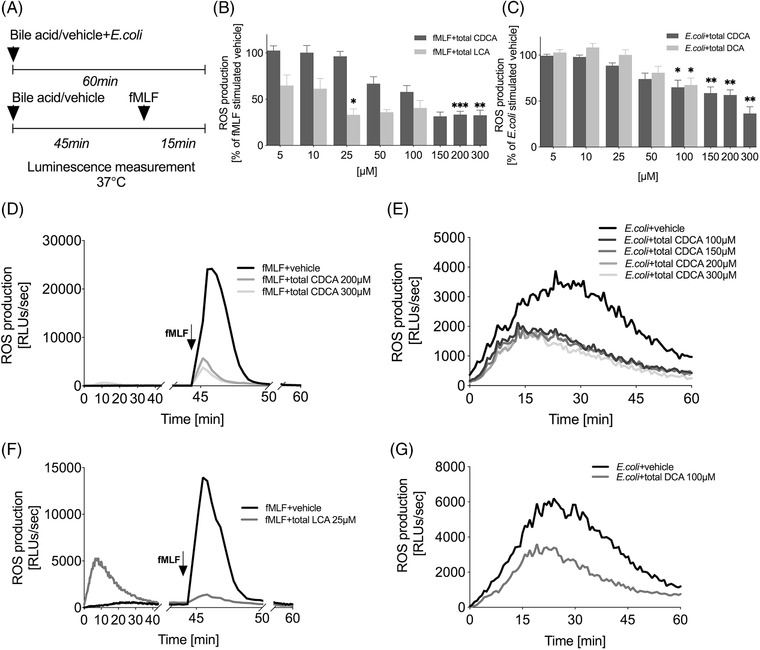
Bile acids inhibit neutrophil ROS production in response to fMLF and *E. coli*. (A) Experimental setup. (B–G) ROS production of isolated healthy donor neutrophils after treatment with bile acid/vehicle and stimulation with (B, D, and F) fMLF (1.45 μM) or (C, E, and G) *E. coli* (40 bacteria/cell). (D–G) Representative examples and (B and C) total ROS production over time. Total ROS data were normalised and compared to vehicle‐treated (PBS or different concentrations of DMSO) stimulated control and represent a minimum of four separate experiments. Data are presented for bile acids, which significantly influenced ROS production; error bars, SEM; *p < .05,**p < .01,***p < .001 (unpaired *t*‐test). CDCA, chenodeoxycholic acid; DCA, deoxycholic acid; DMSO, dimethyl sulfoxide; fMLF, N‐formyl‐met‐leu‐phe; LCA, lithocholic acid; PBS, phosphate‐buffered saline; RLUs, relative light units; ROS, reactive oxygen species; SEM, standard error of the mean

Total CDCA, its unconjugated and conjugated forms individually, as well as total DCA caused a decrease in ROS production in response to *E. coli* (Figure 2C,E,G, Figure S13D,E). DCA has previously been described to inhibit neutrophil chemotaxis and calcium mobilisation and is also thought to be able to inhibit FPR1 signalling.^9^ There were no significant changes in ROS production after the treatment with total cholic acid (CA) and total UDCA (Figure S12A–C). Reduction in ROS production in response to fMLF and *E. coli* caused by BAs may contribute to an explanation for the reduced ROS production, which has been shown previously in neutrophils from cirrhotic patients.^10^

We also tested if treatment with BAs influence phagocytosis in vitro (Figure 3A). ‘BA mix’ did not significantly affect neutrophil phagocytosis (Figure 3B); however, total CDCA and total DCA (but neither their unconjugated and conjugated forms individually, nor total CA, total UDCA and total LCA) significantly reduced phagocytosis of *E. coli* (Figure 3C,D; Figures S15A,B and S16A–C). The inhibitory effects of total CDCA and total DCA were reversible – neutrophil phagocytosis was not inhibited anymore after the BAs were washed out from the assay medium (Figure 3E–G).

**FIGURE 3:**
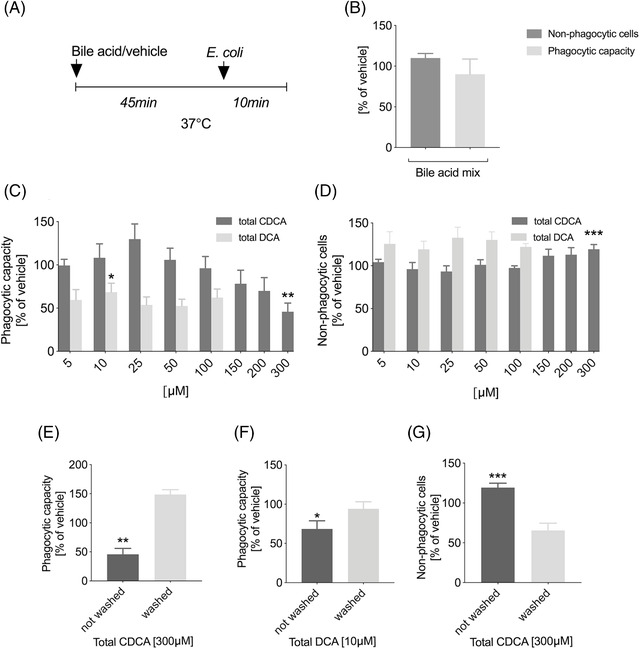
Total CDCA and total DCA inhibit neutrophil phagocytosis. (A) Experimental setup. (B–G) 5 × 10^5^ isolated healthy donor neutrophils were pre‐incubated with bile acids and then allowed to phagocytose 4 × 10^7^ *E. coli*. (E–G) Bile acids were or were not washed out following pre‐incubation with the cells for 45 min. Phagocytic capacity (B, C, E, and F) and percentage of non‐phagocytic neutrophils (B, D, and G) were measured by flow cytometry. The responses were normalised to the response of vehicle‐treated (PBS or different concentrations of DMSO) neutrophils. A minimum of four separate experiments are combined in these graphs; error bars, SEM; *p < .05,**p < .01,***p < .001 (unpaired *t*‐test). CDCA, chenodeoxycholic acid; DCA, deoxycholic acid; DMSO, dimethyl sulfoxide; PBS, phosphate‐buffered saline; SEM, standard error of the mean

In conclusion, we performed an extensive analysis of neutrophil function and serum BA composition in a large cohort of cirrhotic patients. We show that BAs are associated with neutrophil dysfunction in the clinical data from cirrhotic patients, as well as in the experiments where we directly treated neutrophils with BAs. These findings should be validated in patients with more severe stages of cirrhosis, and the molecular mechanisms of the observed BA effects on neutrophil function need to be explored in further studies. The reversibility of neutrophil dysfunction caused by BAs, which we observed, suggests that therapeutical modulation of serum BA profile toward a ‘healthy’ composition could potentially not only prevent neutrophil dysfunction but also restore already impaired neutrophil function in cirrhotic patients. This in turn will allow reducing bacterial infections and associated morbidity and mortality in liver cirrhosis. BA profile modulation may be achieved by oral intake of BAs or modified BAs and by targeting the gut microbiome involved in BA metabolism.

## CONFLICT OF INTEREST

All authors have nothing to disclose.

## FUNDING INFORMATION

Austrian Science Fund FWF (W1241) and the Medical University Graz through the PhD Program Molecular Fundamentals of Inflammation (DK‐MOLIN); Medical Research Council (MRC) MR/S008020/1.

## Supporting information

Supplementary material
