# Supplementary material for: Serum bile acids in liver cirrhosis promote neutrophil dysfunction
Source: Clin Transl Med. 2022 Feb 27;12(2):e735. doi: 10.1002/ctm2.735 (PMC8882235; doi:10.1002/ctm2.735)
Supplement: Supplementary file 1 — Supplementary material [file CTM2-12-e735-s001.docx]

**Supplementary material**

**Serum bile acids in liver cirrhosis promote neutrophil dysfunction**

Irina Balazs^1,2^, Angela Horvath^1,2^, Bettina Leber^3^, Nicole Feldbacher^1,2^, Wolfgang Sattler^4,5^, Florian Rainer^1^, Günter Fauler^6^, Sonja Vermeren^7^, Vanessa Stadlbauer^1,2,^*

^1^ Department of Internal Medicine, Division of Gastroenterology and Hepatology, Medical University of Graz, Graz, Austria

^2^ Center for Biomarker Research in Medicine (CBmed), Graz, Austria

^3^ Department of Surgery, Division of Transplantation Surgery, Medical University of Graz, Graz, Austria

^4^ Gottfried Schatz Research Center (for Cell Signaling, Metabolism and Aging), Division of Molecular Biology and Biochemistry, Medical University of Graz, Graz, Austria

^5^ Center for Explorative Lipidomics, BioTechMed Graz, Graz, Austria

^6^ Clinical Institute of Medical and Chemical Laboratory Diagnostics, Medical University of Graz, Graz, Austria

^7^ Centre for Inflammation Research, University of Edinburgh, Edinburgh, UK

***Corresponding author:** Vanessa Stadlbauer; Department of Internal Medicine, Division of Gastroenterology und Hepatology, Medical University of Graz, Auenbruggerplatz 15, 8036 Graz; phone: +43/ 316/ 385 82282; fax: +43/ 316/ 385-595-82282; e-mail: [vanessa.stadlbauer@medunigraz.at](mailto:vanessa.stadlbauer@medunigraz.at)

**Table of contents:**

*Supplementary Material and Methods*

*Supplementary Fig.1*

*Supplementary Fig.2*

*Supplementary Fig.3*

*Supplementary Fig.4*

*Supplementary Fig.5*

*Supplementary Fig.6*

*Supplementary Fig.7*

*Supplementary Fig.8*

*Supplementary Fig.9*

*Supplementary Fig.10*

*Supplementary Fig.11*

*Supplementary Fig.12*

*Supplementary Fig.13*

*Supplementary Fig.14*

*Supplementary Fig.15*

*Supplementary Fig.16*

*Supplementary Table 1*

*Supplementary Table 2*

*Supplementary Table 3*

*Supplementary Table 4*

*Supplementary Table 5*

*Supplementary Table 6*

*Supplementary Table 7*

*Supplementary Table 8*

**Supplementary material and methods**

**Materials**

All reagents, including BAs, were obtained from Sigma-Aldrich (St. Louis, Missouri, USA) unless stated otherwise. Glycolithocholic acid (GLCA) sodium salt was ordered from Santa Cruz Biotechnology (Dallas, Texas, USA). PBS and Roswell Park Memorial Institute (RPMI) 1640 containing L-glutamine were from Gibco^TM^ Thermo Fisher Scientific (Waltham, Massachusetts, USA). Tauroursodeoxycholic acid, taurocholic acid, glycocholic acid, taurochenodeoxycholic acid, glycochenodeoxycholic acid, DCA, taurodeoxycholic acid, glycodeoxycholic acid were diluted in sterile distilled water; UDCA, glycoursodeoxycholic acid, CA, CDCA, LCA, taurolithocholic acid, GLCA were diluted in DMSO.

**Human samples**

Baseline blood samples from 109 cirrhotic patients recruited into different studies (ClinicalTrials.gov identifiers: NCT01607528, NCT02545335, NCT02545309; ethic vote numbers: 23-096 ex 10/11, 25-006 ex 12/13, 26-569 ex 13/14) and 21 healthy controls were analysed for BA concentrations and neutrophil function. Patients were recruited from the outpatient clinic at the Department of Gastroenterology and Hepatology or the Department of Transplantation Surgery at the University Hospital of Graz between 2012 and 2015. Studies were approved by the Medical University of Graz Institutional Review Board and performed according to the Declaration of Helsinki after patients provided written informed consent. For this analysis patients were included when they were stable outpatients with clinical/radiological/histological evidence of liver cirrhosis of any cause. Patients were not included into the study if they had active infection, active alcohol abuse, organ failure, malignancy, pregnancy, if they used immunomodulating medications or antibiotics within the previous month or were treated with UDCA or if they were under 18 years old. None of the patients received any study specific intervention at the time of blood sampling for this analysis. Blood sampling from healthy donors for the experiments with BAs was performed after donors had provided written informed consent and was approved by the Institutional Review Board of the Medical University of Graz (ethic vote number: 23-096 ex 10/11) and the local Lothian Research Ethics Committee (AMREC 15-HV-013). Healthy controls did not have any known acute or chronic diseases, active infections, pregnancy or medications intake.

**Serum BAs**

Serum BA concentrations in patients and healthy controls were determined by high performance liquid chromatography - high-resolution mass spectrometry as previously described ^1^.

**Neutrophil ROS production and phagocytic capacity measured in whole blood (patients’ samples)**

ROS production in response to different stimuli by neutrophils in freshly taken whole blood was analysed using the Phagoburst^TM^ kit (Glycotope, Heidelberg, Germany) Percentage of neutrophils producing ROS and their intracellular ROS pool were measured. Phagocytic capacity of neutrophils in freshly taken whole blood was determined using the Phagotest^TM^ kit (Glycotope). Phagocytic capacity of neutrophils and amount of non-phagocytic neutrophils were calculated as described ^2^. Phagocytic capacity of FITC-labelled *E. coli* was calculated as a weighted geometric mean fluorescence intensity of phagocytic neutrophil populations. Non-phagocytic neutrophils were defined as percentage of FITC-negative neutrophils, which did not engulf any bacteria. Flow cytometry was performed using an LSRII flow cytometer (BD Biosciences, San Jose, California, USA) with BD FACS Diva^TM^ 6.2 software and analysis was performed using FlowJo^TM^ V10 (BD Biosciences).

**Human neutrophil isolation for *in vitro* studies**

Human neutrophils were isolated from freshly taken healthy venous donor blood by dextran (Serva, Heidelberg, Germany) sedimentation and discontinuous Percoll (Cytiva, Marlborough, Massachusetts, USA) gradient as previously described ^3^. 36ml of whole blood drawn with the 21G butterfly blood collection set and 60ml syringe was gently mixed with 4 ml warm sodium citrate 3.8% solution. 40ml in total was centrifuged at 350xg for 20 min at room temperature (RT). Supernatant was carefully collected and discarded. 6 ml of 6% dextran solution were added to the tube with pelleted cells and 0.9% sodium chloride was added up to 50 ml and the tube was gently rolled to mix the content. Suspension was incubated for 30 min at RT. Supernatant was collected and centrifuged (350xg, 6 min, RT). Supernatant was discarded, cell pellet was resuspended in 55% Percoll and overlaid on a 81%/70% Percoll gradient. After centrifugation (720xg, 20 min, RT) polymorphonuclear cell (PMN) layer was removed to another tube and washed twice with 50 ml of 1x Dulbecco’s phosphate buffered saline (DPBS) without Ca2+ and Mg2+ (230xg, 6 min, RT) and resuspended in assay specific medium. Neutrophils were counted with TC20^TM^ Automated Cell Counter and their viability was determined with trypan blue exclusion staining. All the reagents were sterile to keep cells intact. The method results in neutrophil purity >95% according to Diff-Quik-stained cytocentrifuge preparations.

**Cytotoxicity of BAs**

Freshly isolated human neutrophils were treated with BAs at the indicated concentrations for one hour at 37°C. Propidium iodide was added to the final concentration 1 µg/ml immediately prior to analysis. Data were recorded using a CytoFLEX LX flow cytometer (Beckman Coulter, Brea, California, USA) in combination with CytExpert 2.3 software (Beckman Coulter). FlowJo^TM^ V10 software (BD Biosciences) was used to analyse the fraction of propidium iodide positive cells.

**Detection of ROS in isolated neutrophils**

ROS production was measured indirectly using chemiluminescence in 5x10^5^ freshly isolated neutrophils per well at 37^o^C in luminescence-grade 96 well plates (Nunc, Thermo Fisher Scientific or Greiner Bio-One, Kremsmünster, Austria) in a Cytation plate reader (BioTek, Swindon, UK) or a Lumistar Omega luminescence microplate reader (BMG Labtech, Offenburg, Germany) as previously described ^3^. Neutrophils were treated with physiologically relevant concentrations of BAs (Supplementary Table 1, 2) or vehicle (PBS or DMSO as appropriate, depending on the stimulus and BA used), incubated with 150 µM luminol and 18.75 U/ml horseradish peroxidase for analysis of total ROS (sum of intracellular and extracellular ROS), and stimulated with N-formyl-Met-Leu-Phe (fMLF) or heat-killed, serum-opsonised *E. coli* as indicated. Data output was recorded as relative light units.

**Phagocytic capacity of isolated neutrophils**

Freshly isolated human neutrophils were resuspended in RPMI 1640 medium containing L-glutamine and 10% of autologous serum and treated with BAs or vehicle (RPMI or DMSO as appropriate, depending on the BA used) for 45 min at 37°C. The Phagotest^TM^ kit (Celonic, Basel, Switzerland) was used to determine phagocytic capacity of isolated neutrophils according to the manufacturer’s instructions. 100 µl of neutrophil suspension containing 5x10^5^ cells were used per test tube. Data were recorded using a CytoFLEX LX flow cytometer (Beckman Coulter) with CytExpert 2.3 software (Beckman Coulter) and analysis was performed using FlowJo^TM^ V10 (BD Biosciences). Phagocytic capacity and non-phagocytic neutrophils amount were calculated as described ^2^.

**Statistical analysis**

Where data met the assumption of normality according to the Shapiro-Wilk normality test, unpaired t-test was used; otherwise, Mann-Whitney U test was used. For a comparison of more than two groups of independent samples, one-way analysis of variance with Tukey post hoc test was used for normally distributed data or Kruskal-Wallis test with Bonferroni or Dunn’s multiple comparisons test for non-parametric analysis. Pearson’s chi-squared test was used to compare groups of categorical data. Outliers were detected by the ROUT method (Q=1%). Spearman’s correlation coefficient with Benjamini-Hochberg adjustment for multiple tests, partial Spearman correlation coefficient and multiple linear regression were calculated to assess the variables relationships. Where assumptions of linear regression were violated, log10 or cube root transformations were performed and outliers were removed. An analysis of similarity test based on Bray-Curtis distance with Bonferroni corrected p-values (999 permutations) was used to assess the significance of the groups’ differences, visualized by non-metric multidimensional scaling based on the Bray-Curtis distance. The similarity percentages breakdown procedure was used to evaluate each variable contribution to the groups’ dissimilarities. Missing values were omitted. *p* < 0.05 was considered statistically significant. Analysis was performed in IBM SPSS Statistics V25.0 (IBM, Armonk, New York, USA), GraphPad Prism V9 (GraphPad Software, San Diego, California, USA), Past V4.03 ^4^ and R V4.0.3 software (“psych”, “ppcor”, “vegan”, “ggplot2”, “kader” packages ^5-9^) ^10^ in an integrated development environment RStudio V1.3.1093 ^11^.

**
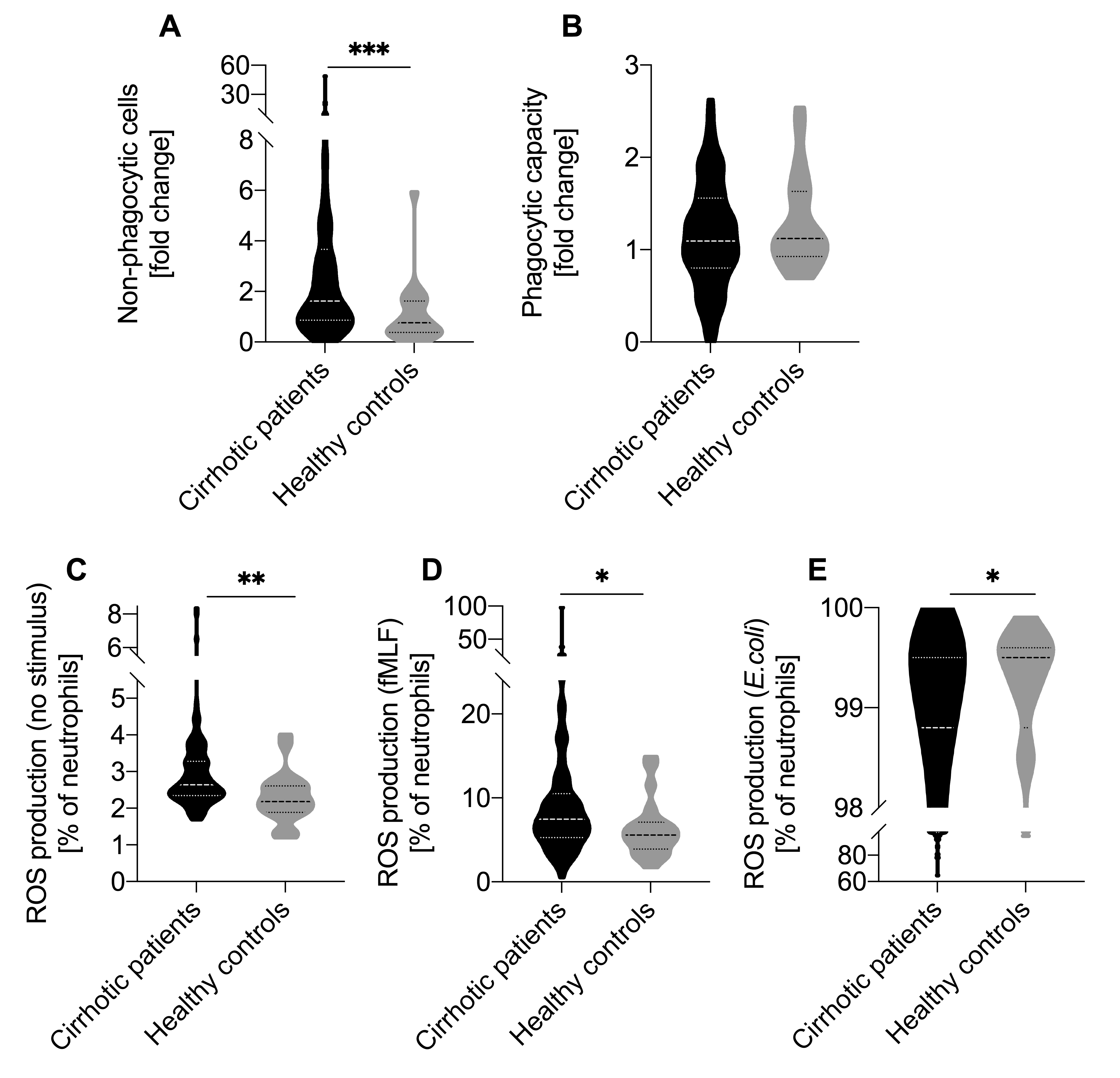
**

**Supplementary Fig.1. Defective neutrophil phagocytosis and ROS production in whole blood of cirrhotic patients.** (A, B) Phagocytosis of *E. coli* (4x10^7^ bacteria/100µl blood). (A) Percentage of non-phagocytic neutrophils and (B) neutrophil phagocytic capacity normalised to *E. coli* batch; (C-E) ROS production. Percentage of neutrophils, which produced ROS (C) without any stimulus or after 10 min of (D) fMLF (0.8µM) or (E) *E. coli* (2-4x10^7^ bacteria/100µl blood) stimulation. Truncated violin plots show the frequency distribution of measured parameters, the broken line indicates median, dotted lines indicate quartiles; cirrhotic patients n=108, healthy controls n=21; *p < 0.05; **p < 0.01; ***p < 0.001 (Mann-Whitney U test). ROS: reactive oxygen species; fMLF: N-formyl-met-leu-phe.

**Supplementary Fig. 2. Intracellular ROS amount produced by neutrophils in whole blood is not different in cirrhotic patients compared to healthy controls.** (A-C) Intracellular ROS pool, defined as GMFI of neutrophils, which produced ROS (A) without a stimulus or after 10 min of (B) fMLF (0.8µM) or (C) *E.coli* (2-4x10^7^ bacteria/100µl blood) stimulation. Truncated violin plots show the frequency distribution of measured parameters, the broken line indicates median, dotted lines indicate quartiles. cirrhotic patients n=108, healthy controls n=21; Statistical analysis was by Mann-Whitney U test (A-C). ROS: reactive oxygen species; GMFI: geometric mean fluorescence intensity; fMLF: N-formyl-met-leu-phe.

**Supplementary Fig. 3. Serum bile acid composition is altered in cirrhotic patients.** (A-G) Serum bile acids in 109 cirrhotic patients and 21 healthy controls. (A, B) Multivariate analysis of bile acid (A) concentrations and (B) relative abundances; (C) Fold change of the patients’ samples compared to the healthy controls; (D-G) Relative abundance of different bile acid groups; (A, B) NMMDS and ANOSIM, ellipses: 95% CI; (C) vertical line is at zero (no change), circles: full – significant; empty – n.s. (Mann-Whitney U test/unpaired t-test); (D-G) truncated violin plots: frequency distribution, the broken line: median, dotted lines: quartiles, ***p < 0.001 (Mann-Whitney U test). ANOSIM: analysis of similarity; CA: cholic acid; TCA: taurocholic acid; GCA: glycocholic acid; CDCA: chenodeoxycholic acid; TCDCA: taurochenodeoxycholic acid; GCDCA: glycochenodeoxycholic acid; DCA: deoxycholic acid; TDCA: taurodeoxycholic acid; GDCA: glycodeoxycholic acid; LCA: lithocholic acid; TLCA: taurolithocholic acid; GLCA: glycolithocholic acid; UDCA: ursodeoxycholic acid; TUDCA: tauroursodeoxycholic acid; GUDCA: glycoursodeoxycholic acid.

**Supplementary Fig. 4. Contribution of different bile acids into cirrhotic patient and healthy control groups’ dissimilarity.** (A, B) The similarity percentages breakdown (SIMPER) analysis was performed to analyse contribution of different bile acids into the dissimilarity in bile acid (A) concentrations and (B) relative abundances between the groups of cirrhotic patients and healthy controls. Bars indicate contribution of each bile acid (%). CA: cholic acid; TCA: taurocholic acid; GCA: glycocholic acid; CDCA: chenodeoxycholic acid; TCDCA: taurochenodeoxycholic acid; GCDCA: glycochenodeoxycholic acid; DCA: deoxycholic acid; TDCA: taurodeoxycholic acid; GDCA: glycodeoxycholic acid; ; LCA: lithocholic acid; TLCA: taurolithocholic acid; GLCA: glycolithocholic acid; UDCA: ursodeoxycholic acid; TUDCA: tauroursodeoxycholic acid; GUDCA: glycoursodeoxycholic acid.

**
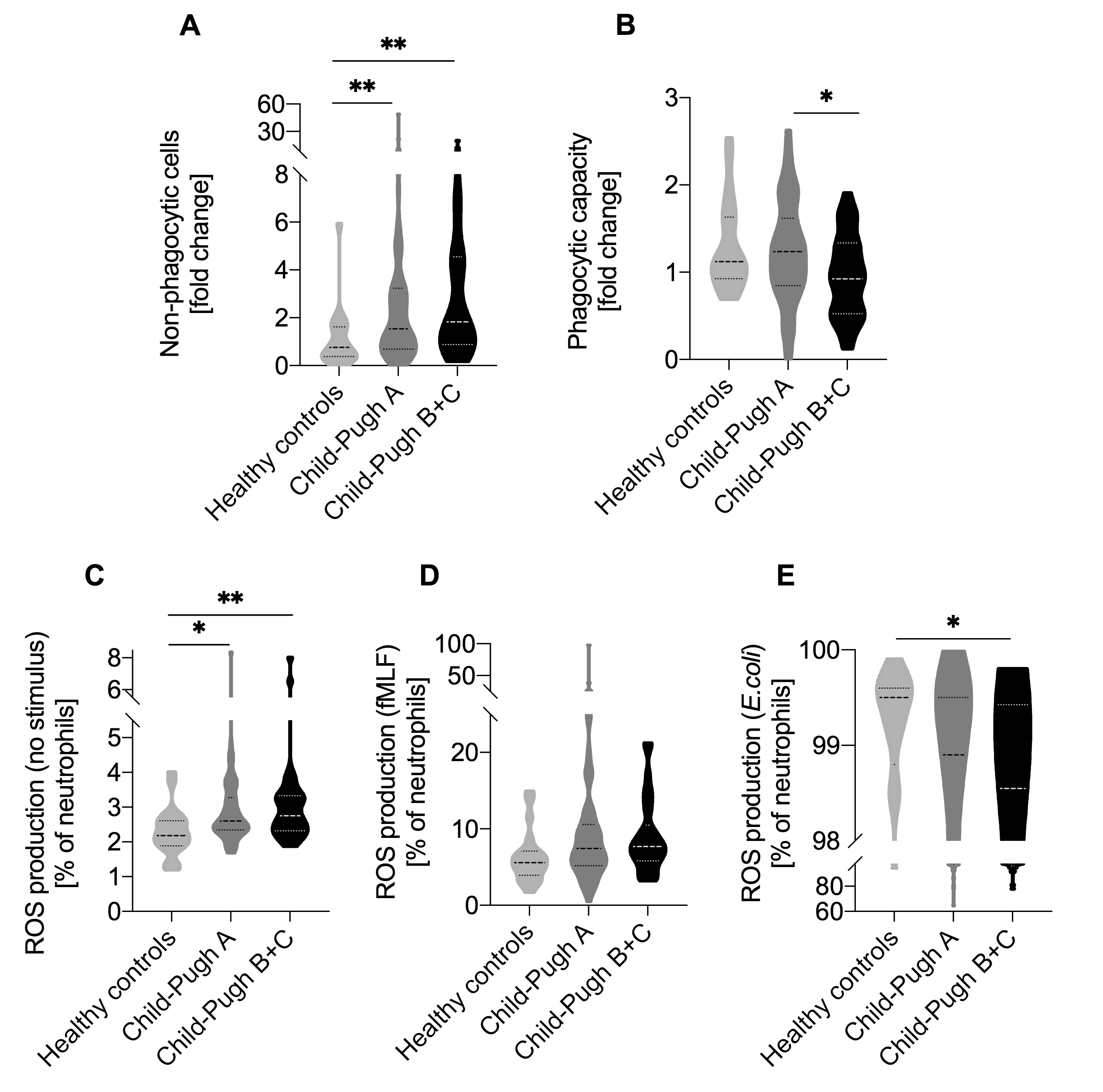
**

**Supplementary Fig. 5. Neutrophil function in whole blood varies between the groups of liver cirrhosis severity.** (A-B) Phagocytosis of *E.coli* (4x10^7^ bacteria/100µl blood). (A) Percentage of non-phagocytic neutrophils and (B) neutrophil phagocytic capacity normalised to *E.coli* batch; (C-E) ROS production. Percentage of neutrophils, which produced ROS (C) without a stimulus or after 10 min of (D) fMLF (0.8µM) or (E) *E.coli* (2-4x10^7^ bacteria/100µl blood) stimulation. Truncated violin plots show the frequency distribution of measured parameters, the broken line indicates median, dotted lines indicate quartiles; *p < 0.05; **p < 0.01 (Kruskal-Wallis test with Dunn’s multiple comparisons test). ROS: reactive oxygen species; fMLF: N-formyl-met-leu-phe.


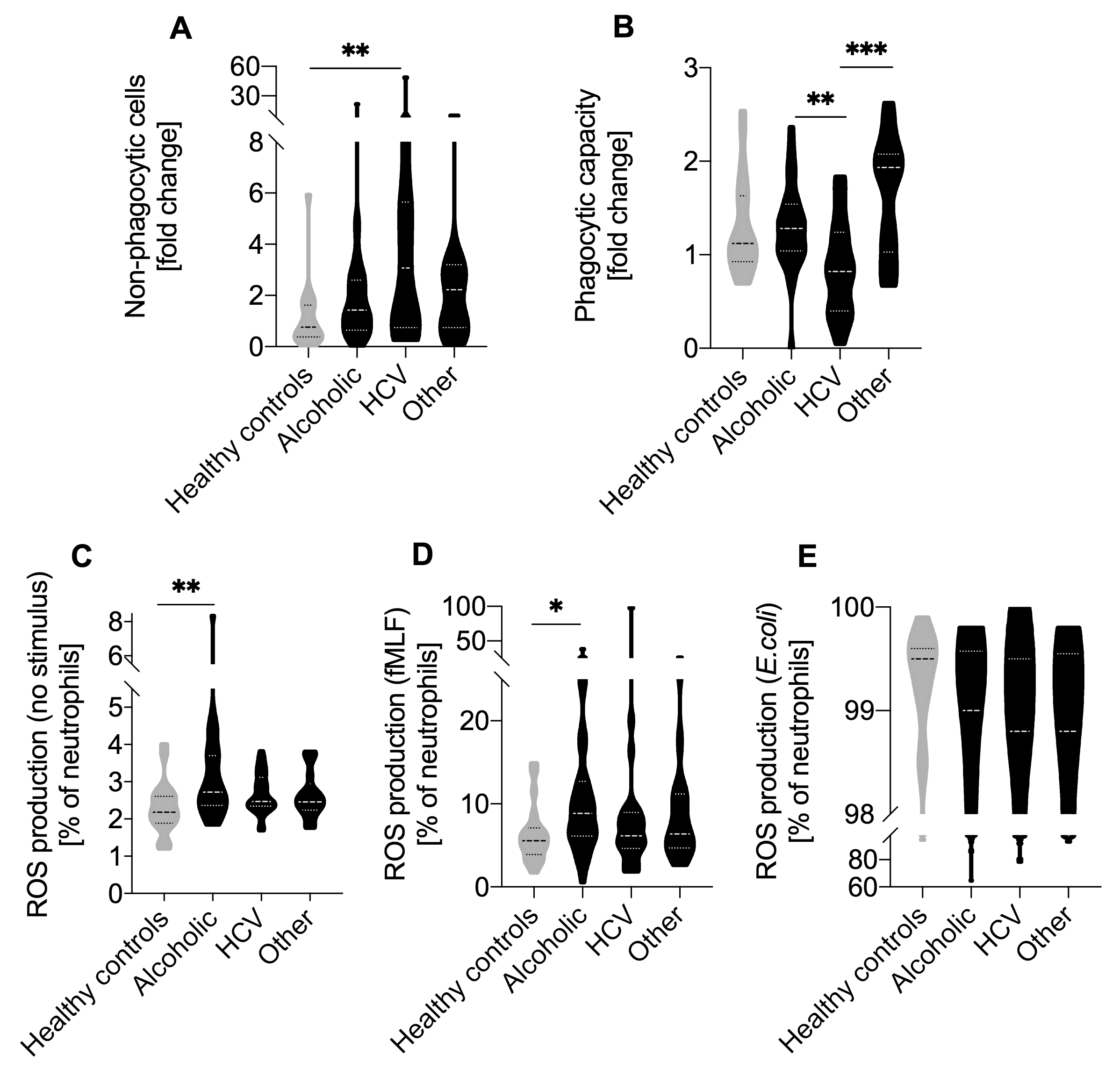


**Supplementary Fig. 6. Neutrophil function in whole blood varies between the groups of liver cirrhosis aetiology.** (A, B) Phagocytosis of *E.coli* (4x10^7^ bacteria/100µl blood). (A) Percentage of non-phagocytic neutrophils and (B) neutrophil phagocytic capacity normalised to *E.coli* batch; (C-E) ROS production. Percentage of neutrophils, which produced ROS (C) without a stimulus or after 10 min of (D) fMLF (0.8µM) or (E) *E.coli* (2-4x10^7^ bacteria/100µl blood) stimulation. Truncated violin plots show the frequency distribution of measured parameters, the broken line indicates median, dotted lines indicate quartiles. Analysed within Child-Pugh A group of patients (n=79); *p < 0.05; **p < 0.01; ***p < 0.001 (Kruskal-Wallis test with Dunn’s multiple comparisons test). ROS: reactive oxygen species; fMLF: N-formyl-met-leu-phe.

**Supplementary Fig. 7. Serum bile acid composition is significantly variable depending on liver cirrhosis severity and aetiology.** (A-D) Serum bile acids in 109 cirrhotic patients and 21 healthy controls. Multivariate analysis of fifteen bile acid (A, C) concentrations and (B, D) relative abundances (% of total bile acids). (C, D) For the analysis of etiology groups only Child-Pugh A patients (n=79) were included. (A-D) Ellipses indicate 95% CI. Statistical analysis was by NNMDS and ANOSIM based on Bray-Curtis distance. ANOSIM: analysis of similarity; NNMDS: non-metric multidimensional scaling.

**Supplementary Fig. 8. Contribution of different bile acids into liver cirrhosis severity groups’ dissimilarity.** (A-F) The similarity percentages breakdown (SIMPER) analysis was performed to analyse contribution of different bile acids into the dissimilarity in bile acid (A-C) concentrations and (D-F) relative abundances between the groups of (A, D) Child-Pugh A and healthy controls, (B, E) Child-Pugh B+C and healthy controls, (C, F) Child-Pugh A and Child Pugh B+C. Bars indicate contribution of each bile acid (%). CA: cholic acid; TCA: taurocholic acid; GCA: glycocholic acid; CDCA: chenodeoxycholic acid; TCDCA: taurochenodeoxycholic acid; GCDCA: glycochenodeoxycholic acid; DCA: deoxycholic acid; TDCA: taurodeoxycholic acid; GDCA: glycodeoxycholic acid; ; LCA: lithocholic acid; TLCA: taurolithocholic acid; GLCA: glycolithocholic acid; UDCA: ursodeoxycholic acid; TUDCA: tauroursodeoxycholic acid; GUDCA: glycoursodeoxycholic acid.

**Supplementary Fig. 9. Contribution of different bile acids into liver cirrhosis aetiology groups’ dissimilarity in bile acid composition between the groups of liver cirrhosis aetiology.** (A-F) The similarity percentages breakdown (SIMPER) analysis was performed to analyse contribution of different bile acids into the dissimilarity in bile acid (A-C) concentrations and (D-F) relative abundances between the groups of (A, D) alcoholic and HCV, (B, E) alcoholic and other, (C, F) HCV and other aetiologies groups. Only Child-Pugh A patients (n=79) were analysed. Bars indicate contribution of each bile acid (%). CA: cholic acid; TCA: taurocholic acid; GCA: glycocholic acid; CDCA: chenodeoxycholic acid; TCDCA: taurochenodeoxycholic acid; GCDCA: glycochenodeoxycholic acid; DCA: deoxycholic acid; TDCA: taurodeoxycholic acid; GDCA: glycodeoxycholic acid; ; LCA: lithocholic acid; TLCA: taurolithocholic acid; GLCA: glycolithocholic acid; UDCA: ursodeoxycholic acid; TUDCA: tauroursodeoxycholic acid; GUDCA: glycoursodeoxycholic acid.

**Supplementary Fig. 10. Changed BA composition in cirrhotic sera associates with dysregulated neutrophil functions.** (A-C) Linear regression analysis of associations between bile acid relative abundances and neutrophil functions in a cohort of cirrhotic patients (n=109). Only significantly correlated variables were analysed with linear regression (partial correlation results: total CDCA relative abundance and phagocytic capacity (r=-0.329, p=0.001), UDCA relative abundance and non-phagocytic neutrophils (r=-0.337, p=0.001), UDCA relative abundance and ROS production (*E. coli*) (r=0.343, p<0.001)).

**Supplementary Fig. 11. Viability of neutrophils after bile acid treatment.** Isolated neutrophils were stained with propidium iodide after 1 hour treatment with bile acids/vehicle (PBS or different concentrations of DMSO) and analysed by flow cytometry. Bars show mean percentage of dead neutrophils (propidium iodide positive) normalised to vehicle and represent a minimum of 4 separate experiments; error bars, SEM; *p<0.05 (unpaired t-test).

**Supplementary Fig. 12. Bile acids and neutrophil ROS production.** (A-C) ROS production after treatment with bile acid/vehicle and stimulation with (A) PBS, (B) fMLF [1.45µM] or (C) *E.coli* [40 bacteria/cell] was measured by chemiluminescence. Total ROS production over time is presented. For ease of viewing, total ROS data were normalised to vehicle-treated (PBS or different concentrations of DMSO) (A) unstimulated or (B, C) stimulated control and represent a mean of a minimum of 4 separate experiments; error bars, SEM. Statistical analysis was by unpaired t-test. ROS: reactive oxygen species; fMLF: N-formyl-met-leu-phe; CA: cholic acid; CDCA: chenodeoxycholic acid; UDCA: ursodeoxycholic acid; DCA: dexycholic acid; LCA: lithocholic acid.

**Supplementary Fig. 13. ROS production in neutrophils treated with individual unconjugated and conjugated forms of bile acids.** (A-E) ROS production after treatment with bile acid/vehicle and stimulation with (A) PBS, (B, C) fMLF [1.45µM] or (D, E) *E.coli* [40 bacteria/cell] was measured by chemiluminescence. Total ROS production over time is presented. For ease of viewing, total ROS data were normalized to vehicle-treated (PBS or different concentrations of DMSO) (A) unstimulated or (B-E) stimulated control and represent a minimum of 4 separate experiments; error bars, SEM; *p < 0.05, **p < 0.01, ***p < 0.001 (unpaired t-test). ROS: reactive oxygen species; fMLF: N-formyl-met-leu-phe, LCA: lithocholic acid; TLCA: taurolithocholic acid; GLC: glycolithocholic acid; CDCA: chenodeoxycholic acid; TCDCA: taurochenodeoxycholic acid; GCDCA: glycochenodeoxycholic acid; DCA: dexycholic acid; TDCA: taurodeoxycholic acid; GDCA: glycodeoxycholic acid.

**Supplementary Fig. 14. ROS production in neutrophils treated with bile acid mix.** (A, B) ROS production after treatment with bile acid (BA) mix/vehicle and stimulation with (A) fMLF [1.45µM] or (B) *E.coli* [40 bacteria/cell] was measured by chemiluminescence. Total ROS production over time is presented. For ease of viewing, total ROS data were normalised to vehicle-treated (PBS) stimulated control and represent a minimum of 4 separate experiments; error bars, SEM. Statistical analysis was by unpaired t-test. ROS: reactive oxygen species; fMLF: N-formyl-met-leu-phe; BAs: bile acids.

**Supplementary Fig. 15. Total CA, total LCA and total UDCA did not significantly influence phagocytosis of neutrophils.** (A, B) 5x10^5^ neutrophils were pre-incubated with bile acids of different concentrations and then allowed to phagocytose 4x10^7^ *E.coli*. (A) Phagocytic capacity and (B) percentage of neutrophils that had not internalized any bacteria were measured by flow cytometry. The responses were normalised to the response of vehicle-treated (different concentrations of DMSO) neutrophils. A minimum of 4 separate experiments are combined in these graphs; error bars, SEM. Statistical analysis was by unpaired t-test. CA: cholic acid; LCA: lithocholic acid; UDCA: ursodeoxycholic acid.

**Supplementary Fig. 16. Phagocytosis in neutrophils treated with individual unconjugated and conjugated forms of bile acids.** (A-C) 5x10^5^ neutrophils were pre-incubated with bile acids of different concentrations and then allowed to phagocytose 4x10^7^ *E.coli*. (A, B) Phagocytic capacity and (C) percentage of neutrophils that had not internalised any bacteria were measured by flow cytometry. The responses were normalised to the response of vehicle-treated (PBS or DMSO of different concentrations) neutrophils. A minimum of 5 separate experiments are combined in these graphs; error bars, SEM; *p < 0.05; **p < 0.01, ***p < 0.001 (unpaired t-test). CDCA: chenodeoxycholic acid; TCDCA: taurochenodeoxycholic acid; GCDCA: glycochenodeoxycholic acid; DCA: dexycholic acid; TDCA: taurodeoxycholic acid; GDCA: glycodeoxycholic acid.

**Supplementary Table 1. Pathophysiological bile acid concentrations used for the treatment of isolated neutrophils.**

| Bile acid | Min/Max concentration in cirrhotic cohort (µM) | Concentration range used for neutrophil treatment (µM) |
| --- | --- | --- |
| CA | 0/23.4 | 5-100 |
| TCA | 0/27.9 |  |
| GCA | 0/72.0 |  |
| CDCA | 0/44.3 | 5-300 |
| TCDCA | 0/131.3 |  |
| GCDCA | 0.3/287.1 |  |
| DCA | 0/3.9 | 5-100 |
| TDCA | 0/7.1 |  |
| GDCA | 0/16.4 |  |
| LCA | 0/0.8 | 5-100 |
| TLCA | 0/0.4 |  |
| GLCA | 0/1.4 |  |
| UDCA | 0/1.4 | 5-100 |
| TUDCA | 0/1.4 |  |
| GUDCA | 0/8.8 |  |

CA: cholic acid; TCA: taurocholic acid; GCA: glycocholic acid; CDCA: chenodeoxycholic acid; TCDCA: taurochenodeoxycholic acid; GCDCA: glycochenodeoxycholic acid; DCA: deoxycholic acid; TDCA: taurodeoxycholic acid; GDCA: glycodeoxycholic acid; LCA: lithocholic acid; TLCA: taurolithocholic acid; GLCA: glycolithocholic acid; UDCA: ursodeoxycholic acid; TUDCA: tauroursodeoxycholic acid; GUDCA: glycoursodeoxycholic acid.

**Supplementary Table 2. Bile acid concentrations and relative abundances in “bile acid mix”.**

| Bile acid | Mean + 2SD concentration in cirrhosis (µM)* | RA in liver cirrhosis (%)* |
| --- | --- | --- |
| TUDCA | 0.684 | 0.2 |
| GUDCA | 3.232 | 2.1 |
| UDCA | 0.532 | 0.7 |
| TCA | 14.702 | 6.0 |
| GCA | 38.413 | 18.6 |
| CA | 5.960 | 1.8 |
| TCDCA | 45.264 | 13.2 |
| GCDCA | 101.329 | 40.5 |
| CDCA | 11.174 | 3.5 |
| TDCA | 2.725 | 1.5 |
| GDCA | 8.501 | 7.2 |
| DCA | 2.034 | 3.8 |
| TLCA | 0.196 | 0.1 |
| GLCA | 0.536 | 0.5 |
| LCA | 0.400 | 0.3 |

RA: relative abundance; CA: cholic acid; TCA: taurocholic acid; GCA: glycocholic acid; CDCA: chenodeoxycholic acid; TCDCA: taurochenodeoxycholic acid; GCDCA: glycochenodeoxycholic acid; DCA: deoxycholic acid; TDCA: taurodeoxycholic acid; GDCA: glycodeoxycholic acid; LCA: lithocholic acid; TLCA: taurolithocholic acid; GLCA: glycolithocholic acid; UDCA: ursodeoxycholic acid; TUDCA: tauroursodeoxycholic acid; GUDCA: glycoursodeoxycholic acid.*Final concentrations and relative abundances, which influenced neutrophils in *in vitro* experiments.

**Supplementary Table 3. Demographic characteristics and liver function parameters of cirrhotic patients and healthy controls.**

| Characteristic | Cirrhotic patients (n=109) | Healthy controls (n=21) | p-value |
| --- | --- | --- | --- |
| Age (years) | 58±13 | 58±11 | *p*=0.790 |
| Sex (Male/Female, n) | 77/32 | 9/12 | *p*=0.014 |
| Aetiology group (Alcoholic/HCV/Other, n) | 54/32/23 | - | - |
| Child-Pugh group (A/B+C, n) | 79/30 | - | - |
| Child-Pugh score | 5±2 | - | - |
| MELD score | 10±6 | - | - |
| AST (U/l) | 49±43 | 23±9 | *p*<0.001 |
| ALT (U/l) | 38±38 | 19±10 | *p*<0.001 |
| GGT (U/l) | 112±160 | 19±15 | *p*<0.001 |
| AP (U/l) | 105±71 | 59±23 | *p*<0.001 |
| Bilirubin (mg/dl) | 1.3±1.3 | 0.5±0.2 | *p*<0.001 |
| INR (ratio) | 1.2±0.3 | 1.0±0.1 | *p*<0.001 |
| Creatinine (mg/dl) | 0.8±0.3 | 0.9±0.3 | *p*=0.218 |
| Albumin (g/dl) | 4.1±0.9 | 4.3±0.2 | *p*=0.071 |
| Neutrophils (x10^9^/l) | 2.8±1.4 | 3.2±1.4 | *p=*0.187 |

Median±IR. ALT: Alanine transaminase; AST: Aspartate transaminase; AP: Alkaline phosphatase; GGT: Gamma-glutamyl transferase; INR: International Normalized Ratio; MELD: Model for End-Stage Liver Disease.

**Supplementary Table 4. Demographic characteristics and liver function parameters of cirrhotic patients depending on liver cirrhosis severity.**

| Characteristic | Child-Pugh A (n=79) | Child-Pugh B+C (n=30) | p-value |
| --- | --- | --- | --- |
| Age (years) | 58±14 | 55.5±8 | *p*=0.134 |
| Sex (Male/Female, n) | 55/24 | 22/8 | *p*=0.704 |
| Aetiology (Alcoholic/HCV/Other, n) | 37/25/17 | 17/7/6 | *p*=0.619 |
| Child-Pugh grade (n) | A –79 | B – 26, C – 4 | - |
| Child-Pugh score | 5±1 | 8±1 | *p*<0.001 |
| MELD score | 8±3 | 15±5 | *p*<0.001 |
| AST (U/l) | 44.5±39 | 59±47 | *p*=0.043 |
| ALT (U/l) | 39±44 | 35±22 | *p*=0.235 |
| GGT (U/l) | 129±169 | 74.5±136 | *p*=0.135 |
| AP (U/l) | 104±62 | 114.5±74 | *p*=0.211 |
| Bilirubin (mg/dl) | 0.9±0.7 | 2.8±3.5 | *p*<0.001 |
| INR (ratio) | 1.2±0.2 | 1.5±0.3 | *p*<0.001 |
| Creatinine (mg/dl) | 0.8±0.3 | 0.8±0.2 | *p*=0.111 |
| Albumin (g/dl) | 4.3±0.7 | 3.2±0.4 | *p*<0.001 |
| Neutrophils (x10^9^/l) | 2.8±1.4 | 2.7±1.5 | *p=*0.820 |

Median±IR. ALT: Alanine transaminase; AST: Aspartate transaminase; AP: Alkaline phosphatase; GGT: Gamma-glutamyl transferase; INR: International Normalized Ratio; MELD: Model for End-Stage Liver Disease.

**Supplementary Table 5. Serum bile acid concentrations, ratios and relative abundances in cirrhotic patients and healthy controls.**

| Bile acid parameter | Cirrhotic patients (n=109) | Healthy controls  (n=21) | p-value |
| --- | --- | --- | --- |
| Total bile acids (µmol/l) | 53.340 (70.620) | 1.748 (1.746) | p<0.001 |
| CA (µmol/l) | 0.742 (2.609) | 0.232 (0.393) | p=0.232 |
| TCA (µmol/l) | 3.692 (5.505) | 0.000 (0.000) | p<0.001 |
| GCA (µmol/l) | 10.460 (13.977) | 0.139 (0.106) | p<0.001 |
| CDCA (µmol/l) | 1.518 (4.828) | 0.341 (0.873) | p=0.001 |
| TCDCA (µmol/l) | 9.400 (17.932) | 0.029 (0.051) | p<0.001 |
| GCDCA (µmol/l) | 22.861 (39.234) | 0.484 (0.551) | p<0.001 |
| DCA (µmol/l) | 0.576 (0.729) | 0.284 (0.316) | p=0.289 |
| TDCA (µmol/l) | 0.619 (1.053) | 0.000 (0.000) | p<0.001 |
| GDCA (µmol/l) | 2.211 (3.145) | 0.141 (0.133) | p<0.001 |
| LCA (µmol/l) | 0.094 (0.153) | 0.000 (0.000) | p<0.001 |
| TLCA (µmol/l) | 0.040 (0.078) | 0.000 (0.000) | p<0.001 |
| GLCA (µmol/l) | 0.130 (0.203) | 0.002 (0.007) | p<0.001 |
| UDCA (µmol/l) | 0.122 (0.205) | 0.042 (0.051) | p=0.150 |
| TUDCA (µmol/l) | 0.146 (0.269) | 0.000 (0.000) | p<0.001 |
| GUDCA (µmol/l) | 0.732 (1.250) | 0.054 (0.089) | p<0.001 |
| CA RA (%) | 1.78 (5.1) | 9.61 (10.93) | p=0.076 |
| TCA RA (%) | 6.04 (5.54) | 0.0 (0.0) | p<0.001 |
| GCA RA (%) | 18.62 (10.05) | 10.73 (5.29) | p<0.001 |
| CDCA RA (%) | 3.52 (5.89) | 11.54 (11.69) | p<0.001 |
| TCDCA RA (%) | 13.16 (10.11) | 1.61 (2.56) | p<0.001 |
| GCDCA RA (%) | 40.49 (12.78) | 27.87 (3.55) | p<0.001 |
| DCA RA (%) | 3.83 (6.47) | 21.21 (13.65) | p<0.001 |
| TDCA RA (%) | 1.50 (1.73) | 0.0 (0.0) | p<0.001 |
| GDCA RA (%) | 7.17 (7.11) | 10.47 (5.57) | p=0.007 |
| LCA RA (%) | 0.32 (0.62) | 0.0 (0.0) | p<0.001 |
| TLCA RA (%) | 0.08 (0.14) | 0.0 (0.0) | p<0.001 |
| GLCA RA (%) | 0.48 (1.01) | 0.07 (0.33) | p<0.001 |
| UDCA RA (%) | 0.65 (1.53) | 4.42 (5.27) | p=0.023 |
| TUDCA RA (%) | 0.22 (0.28) | 0.0 (0.0) | p<0.001 |
| GUDCA RA (%) | 2.14 (2.61) | 2.47 (3.63) | p=0.181 |
| Total CA (µmol/l) | 14.893 (18.161) | 0.371 (0.416) | p<0.001 |
| Total CDCA (µmol/l) | 33.778 (55.114) | 0.854 (1.066) | p<0.001 |
| Total DCA (µmol/l) | 3.405 (4.394) | 0.425 (0.335) | p<0.001 |
| Total LCA (µmol/l) | 0.264 (0.369) | 0.002 (0.007) | p<0.001 |
| Total UDCA (µmol/l) | 1.000 (1.563) | 0.097 (0.125) | p<0.001 |
| Total CA RA (%) | 26.44 (12.33) | 20.34 (9.46) | p=0.033 |
| Total CDCA RA (%) | 57.17 (14.27) | 41.02 (16.48) | p<0.001 |
| Total DCA RA (%) | 12.50 (12.77) | 31.68 (16.35) | p<0.001 |
| Total LCA RA (%) | 0.88 (1.52) | 0.07 (0.33) | p<0.001 |
| Total UDCA RA (%) | 3.01 (3.60) | 6.89 (6.48) | p=0.059 |
| Primary (µmol/l) | 48.671 (68.068) | 1.225 (1.420) | p<0.001 |
| Secondary (µmol/l) | 4.669 (5.191) | 0.523 (0.378) | p<0.001 |
| Secondary to primary (ratio) | 0.250 (0.328) | 0.851 (0.867) | p<0.001 |
| Primary RA (%) | 83.62 (14.63) | 61.35 (18.03) | p<0.001 |
| Secondary RA (%) | 16.39 (14.63) | 38.65 (18.03) | p<0.001 |
| Unconjugated (µmol/l) | 3.051 (7.240) | 0.899 (1.527) | p=0.001 |
| Conjugated (µmol/l) | 50.289 (69.031) | 0.849 (0.861) | p<0.001 |
| Unconjugated to conjugated (ratio) | 0.173 (0.488) | 1.552 (2.106) | p<0.001 |
| Unconjugated RA (%) | 10.10 (13.48) | 46.78 (22.95) | p<0.001 |
| Conjugated RA (%) | 89.90 (13.48) | 53.22 (22.95) | p<0.001 |
| Taurine conjugated (µmol/l) | 13.896 (22.763) | 0.029 (0.051) | p<0.001 |
| Glycine conjugated (µmol/l) | 36.393 (51.253) | 0.820 (0.818) | p<0.001 |
| Taurine to glycine conjugated (ratio) | 0.368 (0.467) | 0.026 (0.038) | p<0.001 |
| Taurine conjugated RA (%) | 22.47 (14.76) | 2.38 (3.41) | p<0.001 |
| Glycine conjugated RA (%) | 77.53 (14.75) | 97.62 (3.41) | p<0.001 |
| Non-12-alpha hydroxylated (µmol/l) | 35.042 (56.432) | 0.953 (1.088) | p<0.001 |
| 12-alpha hydroxylated (µmol/l) | 18.298 (20.309) | 0.795 (0.711) | p<0.001 |
| 12-alpha hydroxylated to non-12-alpha hydroxylated (ratio) | 0.748 (0.521) | 1.475 (1.539) | p=0.001 |
| Non-12-alpha hydroxylated RA (%) | 61.06 (14.19) | 47.98 (15.46) | p<0.001 |
| 12-alpha hydroxylated RA (%) | 38.94 (14.19) | 52.02 (15.46) | p<0.001 |
| Hydrophilic (µmol/l) | 15.893 (18.859) | 0.468 (0.456) | p<0.001 |
| Hydrophobic (µmol/l) | 37.448 (56.243) | 1.281 (1.368) | p<0.001 |
| Hydrophilic to hydrophobic (ratio) | 0.459 (0.287) | 0.405 (0.228) | p=0.307 |
| Hydrophilic RA (%) | 29.45 (11.02) | 27.23 (10.37) | p=0.307 |
| Hydrophobic RA (%) | 70.55 (11.02) | 72.77 (10.37) | p=0.307 |

Mean (SD). RA: relative abundance (% of total bile acids*). CA: cholic acid; TCA: taurocholic acid; GCA: glycocholic acid; CDCA: chenodeoxycholic acid; TCDCA: taurochenodeoxycholic acid; GCDCA: glycochenodeoxycholic acid; DCA: deoxycholic acid; TDCA: taurodeoxycholic acid; GDCA: glycodeoxycholic acid; LCA: lithocholic acid; TLCA: taurolithocholic acid; GLCA: glycolithocholic acid; UDCA: ursodeoxycholic acid; TUDCA: tauroursodeoxycholic acid; GUDCA: glycoursodeoxycholic acid; total CA: CA + TCA + GCA; total CDCA: CDCA + TCDCA + GCDCA; total DCA: DCA + TDCA + GDCA; total LCA: LCA + TLCA + GLCA; total UDCA: UDCA + TUDCA + GUDCA; primary: total CA + total CDCA; secondary: total DCA + total LCA + total UDCA; unconjugated: CA + CDCA + DCA + LCA + UDCA; conjugated: taurine conjugated + glycine conjugated; taurine conjugated: TCA + TCDCA + TDCA + TLCA + TUDCA; glycine conjugated: GCA + GCDCA + GDCA + GLCA + GUDCA; *taurine conjugated RA: % of conjugated; *glycine conjugated RA: % of conjugated; non-12-alpha hydroxylated: total CDCA + total LCA + total UDCA; 12-apha hydroxylated: total CA + total DCA; hydrophilic: total CA + total UDCA; hydrophobic: total CDCA + total DCA + total LCA

**Supplementary Table 6. Serum bile acid concentrations, ratios and relative abundances in patients with different severity of liver cirrhosis and healthy controls.**

| Bile acid parameter | Child-Pugh A (n=79) | Child-Pugh B+C (n=30) | Healthy controls (n=21) | p-value |
| --- | --- | --- | --- | --- |
| Total bile acids (µmol/l) | 29.729 (31.170) | 115.516 (102.186) | 1.748 (1.746) | p<0.001^Δ,&,£^ |
| CA (µmol/l) | 0.658 (2.858) | 0.961 (1.819) | 0.232 (0.393) | p=0.017^&^ p=0.004^£^ |
| TCA (µmol/l) | 2.497 (4.307) | 6.840 (6.986) | 0.000 (0.000) | p<0.001^Δ,&,£^ |
| GCA (µmol/l) | 6.391 (8.468) | 21.172 (19.258) | 0.139 (0.106) | p<0.001^Δ,&,£^ |
| CDCA (µmol/l) | 0.940 (2.733) | 3.038 (7.963) | 0.341 (0.873) | p<0.001^&,£^ |
| TCDCA (µmol/l) | 4.159 (6.392) | 23.200 (28.561) | 0.029 (0.051) | p<0.001^Δ,&,£^ |
| GCDCA (µmol/l) | 11.162 (12.550) | 53.669 (62.884) | 0.484 (0.551) | p<0.001^Δ,&,£^ |
| DCA (µmol/l) | 0.625 (0.768) | 0.446 (0.607) | 0.284 (0.316) | n.s. |
| TDCA (µmol/l) | 0.492 (0.714) | 0.951 (1.613) | 0.000 (0.000) | p<0.001^Δ,&^ |
| GDCA (µmol/l) | 1.928 (2.509) | 2.958 (4.372) | 0.141 (0.133) | p<0.001^Δ^ p=0.002^&^ |
| LCA (µmol/l) | 0.077 (0.121) | 0.137 (0.213) | 0.000 (0.000) | p<0.001^Δ^ p=0.001^&^ |
| TLCA (µmol/l) | 0.024 (0.039) | 0.083 (0.126) | 0.000 (0.000) | p<0.001^&^ p=0.014^Δ^ p=0.046^£^ |
| GLCA (µmol/l) | 0.102 (0.160) | 0.206 (0.277) | 0.002 (0.007) | p<0.001^Δ,&^ |
| UDCA (µmol/l) | 0.109 (0.179) | 0.158 (0.261) | 0.042 (0.051) | n.s. |
| TUDCA (µmol/l) | 0.083 (0.168) | 0.311 (0.393) | 0.000 (0.000) | p<0.001^Δ,&,£^ |
| GUDCA (µmol/l) | 0.482 (0.661) | 1.391 (2.007) | 0.054 (0.089) | p<0.001^Δ,&^ p=0.022^£^ |
| CA RA (%) | 1.71 (5.19) | 1.98 (4.93) | 9.61 (10.93) | n.s. |
| TCA RA (%) | 5.89 (5.84) | 6.44 (4.71) | 0.0 (0.0) | p<0.001^Δ,&^ |
| GCA RA (%) | 18.01 (9.23) | 20.24 (11.98) | 10.73 (5.29) | p=0.001^Δ,&^ |
| CDCA RA (%) | 3.30 (5.05) | 4.10 (7.75) | 11.54 (11.69) | p=0.001^Δ^ p=0.002 ^&^ |
| TCDCA RA (%) | 11.57 (8.81) | 17.36 (12.13) | 1.61 (2.56) | p<0.001^Δ,&^ |
| GCDCA RA (%) | 40.02 (13.07) | 41.75 (12.12) | 27.87 (16.25) | p=0.001^Δ,&^ |
| DCA RA (%) | 4.96 (7.22) | 0.84 (1.63) | 21.21 (13.65) | p<0.001^Δ,&^ p=0.001^£^ |
| TDCA RA (%) | 1.70 (1.83) | 1.00 (1.30) | 0.0 (0.0) | p<0.001^Δ^  p=0.001^&^ |
| GDCA RA (%) | 8.42 (7.22) | 3.88 (5.71) | 10.47 (5.57) | p<0.001^&^ p=0.001^£^ |
| LCA RA (%) | 0.34 (0.67) | 0.26 (0.44) | 0.0 (0.0) | p<0.001^Δ^, p=0.004^&^ |
| TLCA RA (%) | 0.08 (0.15) | 0.08 (0.13) | 0.0 (0.0) | p<0.001^&^ p=0.007^Δ^ |
| GLCA RA (%) | 0.56 (1.16) | 0.27 (0.37) | 0.07 (0.33) | p<0.001^Δ^ p=0.002^&^ |
| UDCA RA (%) | 0.75 (1.68) | 0.39 (1.02) | 4.42 (5.27) | n.s. |
| TUDCA RA (%) | 0.21 (0.28) | 0.25 (0.29) | 0.0 (0.0) | p<0.001^Δ,&^ |
| GUDCA RA (%) | 2.50 (2.90) | 1.19 (1.21) | 2.47 (3.63) | n.s. |
| Total CA (µmol/l) | 9.546 (12.286) | 28.972 (23.223) | 0.371 (0.416) | p<0.001^Δ,&,£^ |
| Total CDCA (µmol/l) | 16.261 (17.634) | 79.907 (86.247) | 0.854 (1.066) | p<0.001^Δ,&,£^ |
| Total DCA (µmol/l) | 3.045 (3.488) | 4.355 (6.157) | 0.425 (0.335) | p<0.001^Δ^ p=0.009^&^ |
| Total LCA (µmol/l) | 0.203 (0.273) | 0.426 (0.519) | 0.002 (0.007) | p<0.001^Δ,&^ |
| Total UDCA( µmol/l) | 0.674 (0.886) | 1.859 (2.436) | 0.097 (0.125) | p<0.001^Δ,&^ p=0.008^£^ |
| Total CA RA (%) | 25.60 (12.46) | 28.65 (11.88) | 20.34 (9.46) | p=0.027^&^ |
| Total CDCA RA (%) | 54.88 (13.99) | 63.20 (13.40) | 41.02 (16.48) | p<0.001^Δ,&^ p=0.020^£^ |
| Total DCA RA (%) | 15.08 (13.36) | 5.71 (7.87) | 31.68 (16.35) | p<0.001^Δ,&,£^ |
| Total LCA RA (%) | 0.98 (1.69) | 0.61 (0.87) | 0.07 (0.33) | p<0.001^Δ^ p=0.001^&^ |
| Total UDCA RA (%) | 3.46 (4.02) | 1.83 (1.73) | 6.89 (6.48) | n.s. |
| Primary (µmol/l) | 25.807 (28.995) | 108.879 (99.094) | 1.225 (1.420) | p<0.001^Δ,&,£^ |
| Secondary (µmol/l) | 3.921 (4.036) | 6.639 (7.141) | 0.523 (0.378) | p<0.001^Δ,&^ |
| Secondary to primary (ratio) | 0.307 (0.362) | 0.100 (0.124) | 0.851 (0.867) | p<0.001^Δ,&,£^ |
| Primary RA (%) | 80.49 (15.22) | 91.85 (8.77) | 61.35 (18.03) | p<0.001^Δ,&,£^ |
| Secondary RA (%) | 19.51 (15.22) | 8.15 (8.79) | 38.65 (18.03) | p<0.001^Δ,&,£^ |
| Unconjugated (µmol/l) | 2.410 (5.921) | 4.739 (9.842) | 0.899 (1.527) | p<0.001^&^ p=0.029^Δ^ p=0.010^£^ |
| Conjugated (µmol/l) | 27.319 (29.778) | 110.778 (100.693) | 0.849 (0.861) | p<0.001^Δ,&,£^ |
| Unconjugated to conjugated (ratio) | 0.190 (0.534) | 0.128 (0.346) | 1.552 (2.106) | p<0.001^Δ,&^ |
| Unconjugated RA (%) | 11.06 (13.49) | 7.57 (13.34) | 46.78 (22.95) | p<0.001^Δ,&^ |
| Conjugated RA (%) | 88.94 (13.49) | 92.44 (13.34) | 53.22 (22.95) | p<0.001^Δ,&^ |
| Taurine conjugated (µmol/l) | 7.255 (10.962) | 31.384 (34.205) | 0.029 (0.051) | p<0.001^Δ,&,£^ |
| Glycine conjugated (µmol/l) | 20.064 (22.032) | 79.395 (76.405) | 0.820 (0.818) | p<0.001^Δ,&,£^ |
| Taurine to glycine conjugated (ratio) | 0.330 (0.428) | 0.467 (0.553) | 0.027 (0.038) | p<0.001^Δ,&^ |
| Taurine conjugated RA (%) | 20.87 (14.18) | 26.67 (15.64) | 2.38 (3.41) | p<0.001^Δ,&^ |
| Glycine conjugated RA (%) | 79.13 (14.18) | 73.33 (15.64) | 97.62 (3.41) | p<0.001^Δ,&^ |
| Non-12-alpha hydroxylated (µmol/l) | 17.137 (18.250) | 82.191 (88.266) | 0.953 (1.088) | p<0.001^Δ,&,£^ |
| 12-alpha hydroxylated (µmol/l) | 12.591 (14.023) | 33.327 (26.167) | 0.795 (0.711) | p<0.001^Δ,&,£^ |
| 12-alpha hydroxylated to non 12-alpha hydroxylated (ratio) | 0.803 (0.540) | 0.604 (0.445) | 1.475 (1.539) | p<0.001 ^&^p=0.022^Δ^ |
| Non-12-alpha hydroxylated RA (%) | 59.32 (14.20) | 65.64 (13.32) | 47.98 (15.46) | p<0.001^&^ p=0.004^Δ^ |
| 12-alpha hydroxylated RA (%) | 40.68 (14.20) | 34.36 (13.33) | 52.02 (15.46) | p<0.001^&^ p=0.004^Δ^ |
| Hydrophilic (µmol/l) | 10.220 (12.680) | 30.831 (23.949) | 0.468 (0.456) | p<0.001^Δ,&,£^ |
| Hydrophobic (µmol/l) | 19.509 (19.384) | 84.687 (87.091) | 1.281 (1.368) | p<0.001^Δ,&,£^ |
| Hydrophilic to hydrophobic (ratio) | 0.444 (0.237) | 0.497 (0.392) | 0.405 (0.228) | n.s. |
| Hydrophilic RA (%) | 29.07 (10.80) | 30.48 (11.71) | 27.23 (10.37) | n.s. |
| Hydrophobic RA (%) | 70.93 (10.80) | 69.53 (11.71) | 72.77 (10.37) | n.s. |

Mean (SD). ^Δ^Significant difference between Child-Pugh A and healthy group, ^&^significant difference between Child-Pugh B+C and healthy controls; ^£^significant difference between Child-Pugh A and Child-Pugh B+C groups. RA: relative abundance (% of total bile acids*). CA: cholic acid; TCA: taurocholic acid; GCA: glycocholic acid; CDCA: chenodeoxycholic acid; TCDCA: taurochenodeoxycholic acid; GCDCA: glycochenodeoxycholic acid; DCA: deoxycholic acid; TDCA: taurodeoxycholic acid; GDCA: glycodeoxycholic acid; LCA: lithocholic acid; TLCA: taurolithocholic acid; GLCA: glycolithocholic acid; UDCA: ursodeoxycholic acid; TUDCA: tauroursodeoxycholic acid; GUDCA: glycoursodeoxycholic acid; total CA: CA + TCA + GCA; total CDCA: CDCA + TCDCA + GCDCA; total DCA: DCA + TDCA + GDCA; total LCA: LCA + TLCA + GLCA; total UDCA: UDCA + TUDCA + GUDCA; primary: total CA + total CDCA; secondary: total DCA + total LCA + total UDCA; unconjugated: CA + CDCA + DCA + LCA + UDCA; conjugated: taurine conjugated + glycine conjugated; taurine conjugated: TCA + TCDCA + TDCA + TLCA + TUDCA; glycine conjugated: GCA + GCDCA + GDCA + GLCA + GUDCA; *taurine conjugated RA: % of conjugated; *glycine conjugated RA: % of conjugated; non-12-alpha hydroxylated: total CDCA + total LCA + total UDCA; 12-apha hydroxylated: total CA + total DCA; hydrophilic: total CA + total UDCA; hydrophobic: total CDCA + total DCA + total LCA

**Supplementary Table 7. Serum bile acid concentrations, ratios and relative abundances in patients with different aetiology of liver cirrhosis and healthy controls.**

| Bile acid parameter | Alcoholic (n=37) | HCV (n=25) | Other (n=17) | Healthy controls (n=21) | p-value |
| --- | --- | --- | --- | --- | --- |
| Total bile acids (µmol/l) | 44.721 (37.258) | 14.760 (11.993) | 19.111 (20.192) | 1.748 (1.746) | p<0.001^Δ,&,£^ p=0.039^®^ |
| CA (µmol/l) | 0.647 (1.723) | 0.087 (0.200) | 1.524 (5.637) | 0.232 (0.393) | p<0.001^®^ |
| TCA (µmol/l) | 4.155 (5.648) | 1.059 (1.462) | 1.000 (1.869) | 0.000 (0.000) | p<0.001^Δ,&,£^ |
| GCA (µmol/l) | 10.448 (10.736) | 2.609 (2.481) | 3.124 (3.047) | 0.139 (0.106) | p<0.001^Δ^ p=0.006^&^ p=0.001^£^ p=0.005^®^ |
| CDCA (µmol/l) | 1.143 (2.903) | 0.322 (0.582) | 1.408 (4.003) | 0.341 (0.873) | p=0.004^Δ^ p=0.034^®^ |
| TCDCA (µmol/l) | 6.592 (8.231) | 2.281 (3.079) | 1.625 (2.545) | 0.029 (0.051) | p<0.001^Δ,&^ p=0.001^£^ |
| GCDCA (µmol/l) | 16.373 (14.389) | 6.293 (4.946) | 6.982 (12.119) | 0.484 (0.551) | p<0.001^Δ,&^ p=0.001^£^ p=0.048° |
| DCA (µmol/l) | 0.694 (0.787) | 0.291 (0.359) | 0.967 (0.992) | 0.284 (0.316) | p=0.026^®^ p=0.013^ |
| TDCA (µmol/l) | 0.751 (0.903) | 0.266 (0.441) | 0.261 (0.258) | 0.000 (0.000) | p<0.001^Δ,£^ p=0.003^&^ |
| GDCA (µmol/l) | 2.751 (3.253) | 1.138 (1.263) | 1.296 (1.235) | 0.141 (0.133) | p<0.001^Δ^ p=0.001^£^  p=0.007^&^ |
| LCA (µmol/l) | 0.092 (0.114) | 0.043 (0.057) | 0.095 (0.184) | 0.000 (0.000) | p<0.001^Δ^ p=0.020^&^ p=0.013^£^ |
| TLCA (µmol/l) | 0.030 (0.041) | 0.019 (0.038) | 0.019 (0.036) | 0.000 (0.000) | p=0.003^Δ^ |
| GLCA (µmol/l) | 0.096 (0.111) | 0.071 (0.098) | 0.157 (0.280) | 0.002 (0.007) | p<0.001^Δ,£^ p=0.002^&^ |
| UDCA (µmol/l) | 0.140 (0.206) | 0.033 (0.050) | 0.153 (0.209) | 0.042 (0.051) | n.s. |
| TUDCA (µmol/l) | 0.142 (0.226) | 0.026 (0.041) | 0.039 (0.067) | 0.000 (0.000) | p<0.001^Δ^ p=0.037^£^ |
| GUDCA (µmol/l) | 0.667 (0.776) | 0.222 (0.204) | 0.459 (0.726) | 0.054 (0.089) | p<0.001^Δ^ p=0.009^&^ p=0.003^£^ |
| CA RA (%) | 1.64 (2.63) | 0.54 (0.92) | 3.56 (10.42) | 9.61 (10.93) | p=0.015^&^ |
| TCA RA (%) | 7.09 (5.83) | 4.90 (4.54) | 4.73 (7.27) | 0.0 (0.0) | p<0.001^Δ,&,£^ |
| GCA RA (%) | 20.43 (7.24) | 14.97 (10.37) | 17.20 (10.36) | 10.73 (5.29) | p<0.001^Δ^ |
| CDCA RA (%) | 2.89 (3.67) | 2.67 (3.87) | 5.10 (8.19) | 11.54 (11.69) | p=0.002^&^ p=0.006^Δ^ |
| TCDCA RA (%) | 12.45 (9.77) | 13.28 (7.73) | 7.13 (6.82) | 1.61 (2.56) | p<0.001^Δ,&^ p=0.025^£^ |
| GCDCA RA (%) | 38.24 (10.41) | 47.26 (11.55) | 33.22 (15.82) | 27.87 (16.25) | p<0.001^&^ p=0.010^ |
| DCA RA (%) | 3.97 (5.74) | 2.86 (3.95) | 10.22 (10.87) | 21.21 (13.65) | p<0.001^Δ,&^ |
| TDCA RA (%) | 1.85 (1.90) | 1.36 (1.79) | 1.85 (1.81) | 0.0 (0.0) | p<0.001^Δ,£^ p=0.001^&^ |
| GDCA RA (%) | 7.96 (6.84) | 7.88 (6.86) | 10.20 (8.58) | 10.47 (5.57) | n.s. |
| LCA RA (%) | 0.22 (0.37) | 0.28 (0.37) | 0.69 (1.24) | 0.0 (0.0) | p=0.002^Δ,&,£^ |
| TLCA RA (%) | 0.07 (0.10) | 0.08 (0.15) | 0.11 (0.21) | 0.0 (0.0) | p=0.007^Δ^ |
| GLCA RA (%) | 0.32 (0.49) | 0.44 (0.58) | 1.23 (2.21) | 0.07 (0.33) | p<0.001^£^ p=0.001^Δ^ p=0.002^&^ |
| UDCA RA (%) | 0.57 (1.03) | 0.38 (0.96) | 1.70 (2.97) | 4.42 (5.27) | p=0.021^&^ |
| TUDCA RA (%) | 0.27 (0.33) | 0.14 (0.21) | 0.17 (0.24) | 0.0 (0.0) | p<0.001^Δ^ p=0.022^&^ p=0.018^£^ |
| GUDCA RA (%) | 2.03 (1.96) | 2.94 (3.84) | 2.89 (3.04) | 2.47 (3.63) | n.s. |
| Total CA (µmol/l) | 15.250 (15.263) | 3.755 (3.721) | 5.649 (6.871) | 0.371 (0.416) | p<0.001^Δ^ p=0.002^£^ p=0.019^&^ p=0.005^®^ |
| Total CDCA (µmol/l) | 24.108 (20.511) | 8.895 (7.859) | 10.015 (14.428) | 0.854 (1.066) | p<0.001^Δ,&^ p=0.001^£^ |
| Total DCA (µmol/l) | 4.196 (4.457) | 1.695 (1.867) | 2.524 (1.781) | 0.425 (0.335) | p<0.001^Δ^ p=0.001^£^ |
| Total LCA (µmol/l) | 0.219 (0.243) | 0.133 (0.164) | 0.271 (0.422) | 0.002 (0.007) | p<0.001^Δ,£^ p=0.001^&^ |
| Total UDCA (µmol/l) | 0.948 (1.057) | 0.282 (0.259) | 0.652 (0.892) | 0.097 (0.125) | p<0.001^Δ^ p=0.005^£^ |
| Total CA RA (%) | 29.16 (9.13) | 20.42 (13.32) | 25.49 (15.24) | 20.34 (9.46) | p=0.014^Δ^ |
| Total CDCA RA (%) | 53.58 (9.77) | 63.22 (13.20) | 45.45 (16.46) | 41.02 (16.48) | p<0.001^&^ p=0.046^®^ p=0.001^ |
| Total DCA RA (%) | 13.77 (11.46) | 12.11 (10.35) | 22.28 (18.46) | 31.68 (16.35) | p<0.001^Δ,&^ |
| Total LCA RA (%) | 0.61 (0.74) | 0.80 (0.86) | 2.03 (3.18) | 0.07 (0.33) | p<0.001^Δ,&,£^ |
| Total UDCA RA (%) | 2.87 (2.85) | 3.46 (4.19) | 4.76 (5.61) | 6.89 (6.48) | n.s. |
| Primary (µmol/l) | 39.357 (34.990) | 12.651 (11.101) | 15.664 (18.832) | 1.225 (1.420) | p<0.001^Δ,&,£^ |
| Secondary (µmol/l) | 5.363 (5.078) | 2.110 (2.097) | 3.447 (2.153) | 0.523 (0.378) | p<0.001^Δ,£^ p=0.024^&^ |
| Secondary to primary (ratio) | 0.246 (0.248) | 0.222 (0.195) | 0.565 (0.595) | 0.851 (0.867) | p<0.001^Δ,&^ |
| Primary RA (%) | 82.75 (13.05) | 83.63 (11.88) | 70.94 (20.27) | 61.35 (18.03) | p<0.001^Δ,&^ |
| Secondary RA (%) | 17.25 (13.05) | 16.37 (11.88) | 29.06 (20.27) | 38.65 (18.03) | p<0.001^Δ,&^ |
| Unconjugated (µmol/l) | 2.716 (4.960) | 0.776 (0.945) | 4.148 (10.325) | 0.899 (1.527) | p=0.004^Δ^ p=0.013^£^ p=0.006^®^ p=0.020^ |
| Conjugated (µmol/l) | 42.005 (35.513) | 13.984 (11.798) | 14.963 (18.375) | 0.849 (0.861) | p<0.001^Δ,&,£^ |
| Unconjugated to conjugated (ratio) | 0.120 (0.160) | 0.078 (0.081) | 0.509 (1.087) | 1.552 (2.106) | p<0.001^Δ^ p=0.038 ^£^ |
| Unconjugated RA (%) | 9.30 (10.20) | 6.73 (6.50) | 21.27 (21.03) | 46.78 (22.95) | p<0.001^Δ,&^ p=0.038^£^ |
| Conjugated RA (%) | 90.70 (10.20) | 93.27 (6.50) | 78.73 (21.03) | 53.22 (22.95) | p<0.001^Δ,&^ p=0.038^£^ |
| Taurine conjugated (µmol/l) | 11.670 (14.119) | 3.651 (4.675) | 2.945 (4.409) | 0.029 (0.051) | p<0.001^Δ,&^ p=0.001^£^ |
| Glycine conjugated (µmol/l) | 30.335 (26.488) | 10.333 (7.683) | 12.018 (15.443) | 0.820 (0.818) | p<0.001^Δ,&,£^ |
| Taurine to glycine conjugated (ratio) | 0.398 (0.557) | 0.291 (0.205) | 0.243 (0.336) | 0.026 (0.038) | p<0.001^Δ,&^ p=0.001^£^ |
| Taurine conjugated RA (%) | 23.09 (15.79) | 20.81 (11.25) | 16.12 (13.93) | 2.38 (3.41) | p<0.001^Δ,&^ p=0.001^£^ |
| Glycine conjugated RA (%) | 76.91 (15.79) | 79.19 (11.25) | 83.88 (13.93) | 97.62 (3.41) | p<0.001^Δ,&^ p=0.001^£^ |
| Non-12-alpha hydroxylated (µmol/l) | 25.275 (21.202) | 9.310 (7.971) | 10.938 (15.077) | 0.953 (1.088) | p<0.001^Δ,&^ p=0.001^£^ |
| 12-alpha hydroxylated (µmol/l) | 19.446 (17.051) | 5.451 (4.869) | 8.173 (7.736) | 0.795 (0.711) | p<0.001^Δ,£^ p=0.001^&^ p=0.007^®^ |
| 12-alpha hydroxylated to non 12-alpha hydroxylated (ratio) | 0.804 (0.339) | 0.556 (0.383) | 1.164 (0.842) | 1.475 (1.539) | p<0.001^&^ p=0.006^£^ p=0.025^®^ |
| Non-12-alpha hydroxylated RA (%) | 57.06 (9.26) | 67.47 (14.32) | 52.24 (17.71) | 47.98 (15.46) | p<0.001^&^ p=0.006^ p=0.025^®^ |
| 12-alpha hydroxylated RA (%) | 42.94 (9.26) | 32.53 (14.32) | 47.76 (17.71) | 52.02 (15.46) | p<0.001^&^ p=0.006^ p=0.025^®^ |
| Hydrophilic (µmol/l) | 16.198 (15.719) | 4.037 (3.745) | 6.300 (6.939) | 0.468 (0.456) | p<0.001^Δ^ p=0.001^£^ p=0.005^&^ p=0.009^®^ |
| Hydrophobic (µmol/l) | 28.522 (22.430) | 10.723 (8.610) | 12.811 (15.248) | 1.281 (1.368) | p<0.001^Δ,&,£^ |
| Hydrophilic to hydrophobic (ratio) | 0.493 (0.194) | 0.342 (0.199) | 0.489 (0.324) | 0.405 (0.228) | p=0.043^®^ |
| Hydrophilic RA (%) | 32.03 (7.99) | 23.88 (11.32) | 30.24 (12.99) | 27.23 (10.37) | p=0.015^®^ |
| Hydrophobic RA (%) | 67.97 (7.99) | 76.12 (11.32) | 69.76 (12.99) | 72.77 (10.37) | p=0.015^®^ |

Mean (SD). Analyzed within Child-Pugh A group of patients. ^Δ^Significant difference between alcoholic and healthy controls group, ^&^significant difference between HCV and healthy controls; ^£^significant difference between other etiologies and healthy controls, ^®^significant difference between alcoholic and HCV group, °significant difference between alcoholic and other etiologies group, ^significant difference between HCV and other aetiologies groups. RA: relative abundance (% of total bile acids*). CA: cholic acid; TCA: taurocholic acid; GCA: glycocholic acid; CDCA: chenodeoxycholic acid; TCDCA: taurochenodeoxycholic acid; GCDCA: glycochenodeoxycholic acid; DCA: deoxycholic acid; TDCA: taurodeoxycholic acid; GDCA: glycodeoxycholic acid; LCA: lithocholic acid; TLCA: taurolithocholic acid; GLCA: glycolithocholic acid; UDCA: ursodeoxycholic acid; TUDCA: tauroursodeoxycholic acid; GUDCA: glycoursodeoxycholic acid; total CA: CA + TCA + GCA; total CDCA: CDCA + TCDCA + GCDCA; total DCA: DCA + TDCA + GDCA; total LCA: LCA + TLCA + GLCA; total UDCA: UDCA + TUDCA + GUDCA; primary: total CA + total CDCA; secondary: total DCA + total LCA + total UDCA; unconjugated: CA + CDCA + DCA + LCA + UDCA; conjugated: taurine conjugated + glycine conjugated; taurine conjugated: TCA + TCDCA + TDCA + TLCA + TUDCA; glycine conjugated: GCA + GCDCA + GDCA + GLCA + GUDCA; *taurine conjugated RA: % of conjugated; *glycine conjugated RA: % of conjugated; non-12-alpha hydroxylated: total CDCA + total LCA + total UDCA; 12-apha hydroxylated: total CA + total DCA; hydrophilic: total CA + total UDCA; hydrophobic: total CDCA + total DCA + total LCA

**Supplementary Table 8. Multiple linear regression to identify, if bile acids are predictive for neutrophil function in cirrhotic patients (n=109).**

Phagocytic capacity ~ Total CDCA relative abundance + Aetiology of cirrhosis + Child-Pugh group +Age + Sex

F(5,102) = 5.85, p <0.001, R^2^ = 0.223

|  | Estimate | Std. Error | t-value | p-value |
| --- | --- | --- | --- | --- |
| Total CDCA relative abundance | -126 | 37 | -3.4 | 0.001 |
| Aetiology of cirrhosis | -0.61 | 6.47 | -0.09 | 0.925 |
| Child-Pugh group | -18.96 | 11.39 | -1.66 | 0.100 |
| Age | -0.52 | 0.55 | -0.94 | 0.351 |
| Sex | -34.75 | 10.95 | -3.18 | 0.002 |

Non-phagocytic neutrophils ~ UDCA relative abundance + Aetiology of cirrhosis + Child-Pugh group +Age + Sex

F(5,101) = 3.83, p = 0.003, R^2^ = 0.159

|  | Estimate | Std. Error | t-value | p-value |
| --- | --- | --- | --- | --- |
| UDCA relative abundance (cube root) | -1.68 | 0.43 | -3.95 | <0.001 |
| Aetiology of cirrhosis | 0.06 | 0.06 | 1.10 | 0.273 |
| Child-Pugh group | 0.09 | 0.10 | 0.89 | 0.374 |
| Age | -0.00 | 0.01 | -0.02 | 0.981 |
| Sex | 0.11 | 0.10 | 1.11 | 0.268 |

ROS production (*E. coli*) ~ UDCA relative abundance+ Aetiology of cirrhosis + Child-Pugh group +Age + Sex

F(5,88) = 2.85, p = 0.020, R^2^ = 0.140

|  | Estimate | Std. Error | t-value | p-value |
| --- | --- | --- | --- | --- |
| UDCA relative abundance (cube root) | 3.48 | 1.27 | 2.75 | 0.007 |
| Aetiology of cirrhosis | -0.03 | 0.17 | -0.19 | 0.853 |
| Child-Pugh group | -0.40 | 0.30 | -1.34 | 0.185 |
| Age | -0.03 | 0.01 | -2.15 | 0.034 |
| Sex | 0.04 | 0.30 | 0.15 | 0.882 |

1. Amplatz B, Zohrer E, Haas C, et al. Bile acid preparation and comprehensive analysis by high performance liquid chromatography-high-resolution mass spectrometry. *Clin Chim Acta.* 2017;464:85-92.

2. Horvath A, Leber B, Schmerboeck B, et al. Randomised clinical trial: the effects of a multispecies probiotic vs. placebo on innate immune function, bacterial translocation and gut permeability in patients with cirrhosis. *Aliment Pharmacol Ther.* 2016;44(9):926-935.

3. Karmakar U, Chu JY, Sundaram K, et al. Immune complex-induced apoptosis and concurrent immune complex clearance are anti-inflammatory neutrophil functions. *Cell Death Dis.* 2021;12(4):296.

4. Hammer Ø, Harper, D.A.T., Ryan, P.D. PAST: Paleontological statistics software package for education and data analysis. *Palaeontologia Electronica.* 2001;4(1):9.

5. *vegan: Community Ecology Package* [computer program]. Version R package version 2.5-52019.

6. *psych: Procedures for Psychological, Psychometric, and Personality Research* [computer program]. Version R package version 1.8.10. Evanston, Illinois: Northwestern University; 2018.

7. *ppcor: Partial and Semi-Partial (Part) Correlation* [computer program]. Version R package version 1.1. 2015.

8. Wickham H. *ggplot2: Elegant Graphics for Data Analysis.* Springer-Verlag New York; 2016.

9. *kader: Kernel Adaptive Density Estimation and Regression* [computer program]. 2017.

10. *R: A Language and Environment for Statistical Computing* [computer program]. Vienna, Austria: R Foundation for Statistical Computing; 2019.

11. *RStudio: Integrated Development Environment for R* [computer program]. Boston, MA: RStudio, PBC; 2020.
